# Pulmonary blastoma treatment response to anti-PD-1 therapy: a rare case report and literature review

**DOI:** 10.3389/fonc.2023.1146204

**Published:** 2023-04-12

**Authors:** Yalin Xie, Ning Su, Chaoxia Li, An Lei, Lei Li, Jianjun Zou, Wencang Cen, Jinxing Hu

**Affiliations:** ^1^ Department of Oncology, Guangzhou Chest Hospital, Guangzhou, China; ^2^ Department of Pathology, Guangzhou Chest Hospital, Guangzhou, China; ^3^ Department of Internal Medicine, Guangzhou Chest Hospital, Guangzhou, China; ^4^ State Key Laboratory of Respiratory Disease, Guangzhou Medical University, Guangzhou, China

**Keywords:** pulmonary blastoma, immunotherapy, sintilimab, checkpoint inhibition, PD-1/PD-L1 axis

## Abstract

Pulmonary blastoma (PB) is a rare and invasive malignancy of the lungs with a poor prognosis. Although the mainstay treatment of PB is surgery, and radiotherapy and chemotherapy have been reported, no standard therapy exists for patients inoperable in advanced stages. Moreover, little is known about driver mutation status and immunotherapy efficacy. This paper presents a male patient diagnosed with classic biphasic PB using CT-guided lung biopsy pathology and immunohistochemistry. The patient’s symptoms included cough, chest pain, shortness of breath, hemoptysis, and hypodynamia. The primary focus of this paper is to discuss the impact of anti-PD-1 immunotherapy on PB. The patient experienced progression-free survival (PFS) of over 27 months following sintilimab second-line anti-PD-1 therapy. The patient has currently survived for nearly 40 months with a satisfactory quality of life.

## Introduction

Pulmonary blastoma (PB) is a rare lung malignancy that accounts for approximately 0.5% of all lung tumors (1). Barnett and Barnard reported it in 1945; since then, only a few hundred cases have been reported worldwide. These tumors originate from pluripotent pulmonary blast cells, and histologically, their morphology is similar to that of lung-fetal tissue, with variable biological behavior. PBs are biphasic malignancies consisting of immature epithelial and/or mesenchymal tissues, both of which are derived from common precursor cells. The unusual histologic pleomorphism of PB makes its diagnosis challenging. However, cytopathology reveals both epithelial and mesenchymal malignant cell characteristics of the tumor, thus helping diagnose PB (2). PB has been reported to occur mostly in adults, largely in young women (3), and 67% to 82% of cases are related to smoking (4). PB in adults usually presents as a large, symptomatic mass that often causes symptoms such as cough, hemoptysis, and chest pain. Clinical treatment of PB depends on its histologic subtype, stage, and clinical presentation and includes surgery, chemotherapy, and radiation therapy. Currently, targeted therapy and immunotherapy are rarely used to treat PB. We report a case of long-term survival in a male patient with locally advanced classical biphasic PB who achieved long-term survival after immunotherapy with sintilimab.

## Case presentation

**Figure 1:**
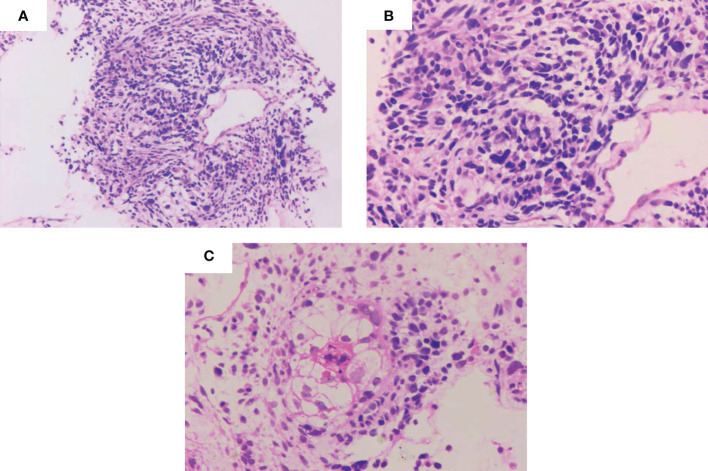
Pathological images of the pulmonary blastoma patient. **(A-C)** Representative images showing spindle-shaped or oval tumor cells with diffuse infiltrative growth, necrosis of limited cartilage components, and loose interstitium. **(A)** Microscopic magnification ×100. **(B, C)** Microscopic magnification ×400.

**Figure 2:**
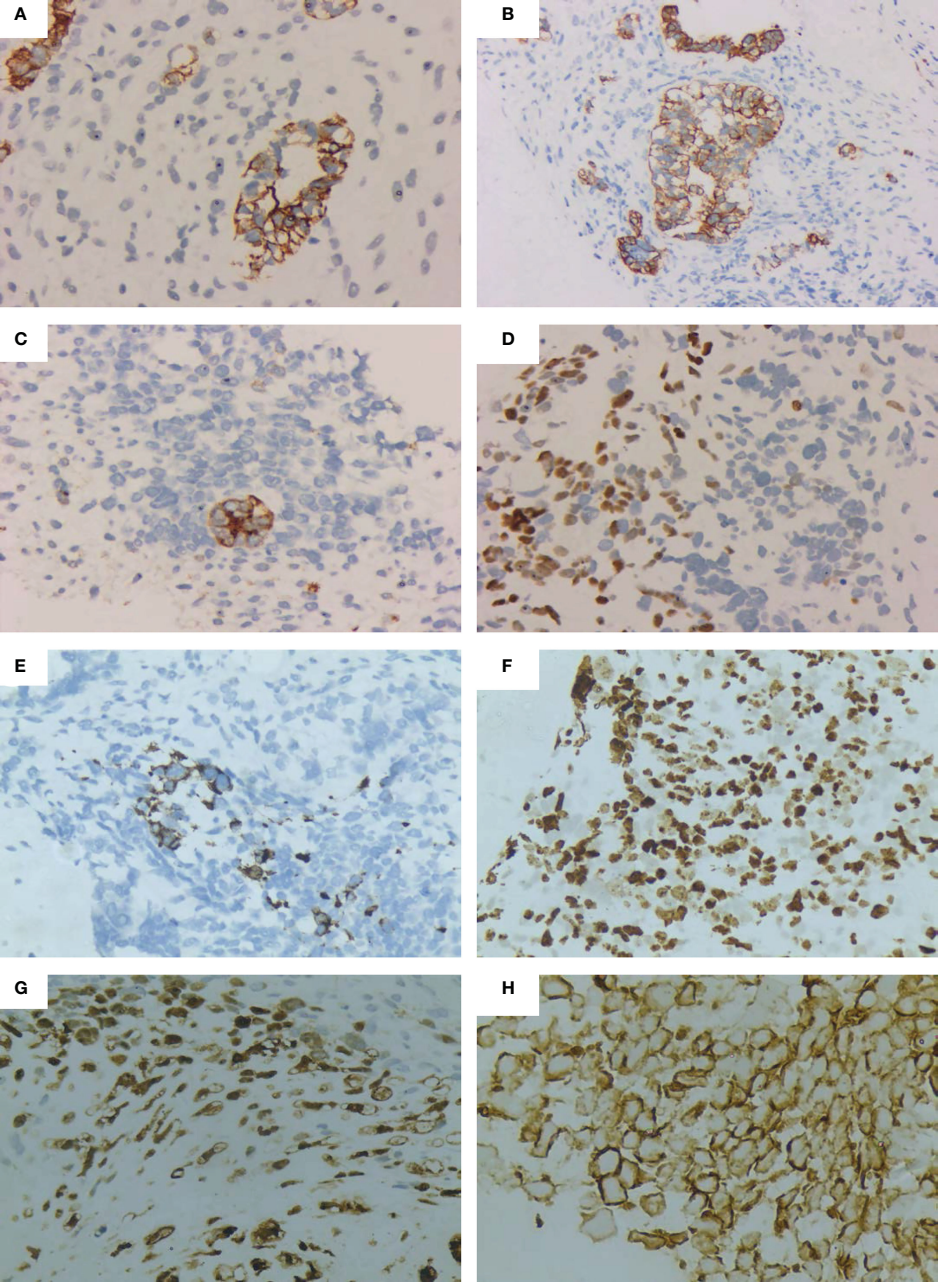
Immunohistochemical images of the pulmonary blastoma patient. **(A)** CK positive(Microscopic magnification×100). **(B)** CK positive (Microscopic magnification×200). **(C)** EMA positive (Microscopic magnification×200). **(D)** TTF-1 positive (Microscopic magnification×200). **(E)** Desmin positive (Microscopic magnification×200). **(F)** Ki-67 positive (Microscopic magnification×200). **(G)** S-100 positive (Microscopic magnification×200). **(H)** Vimentin positive (Microscopic magnification×400).

**Figure 3:**
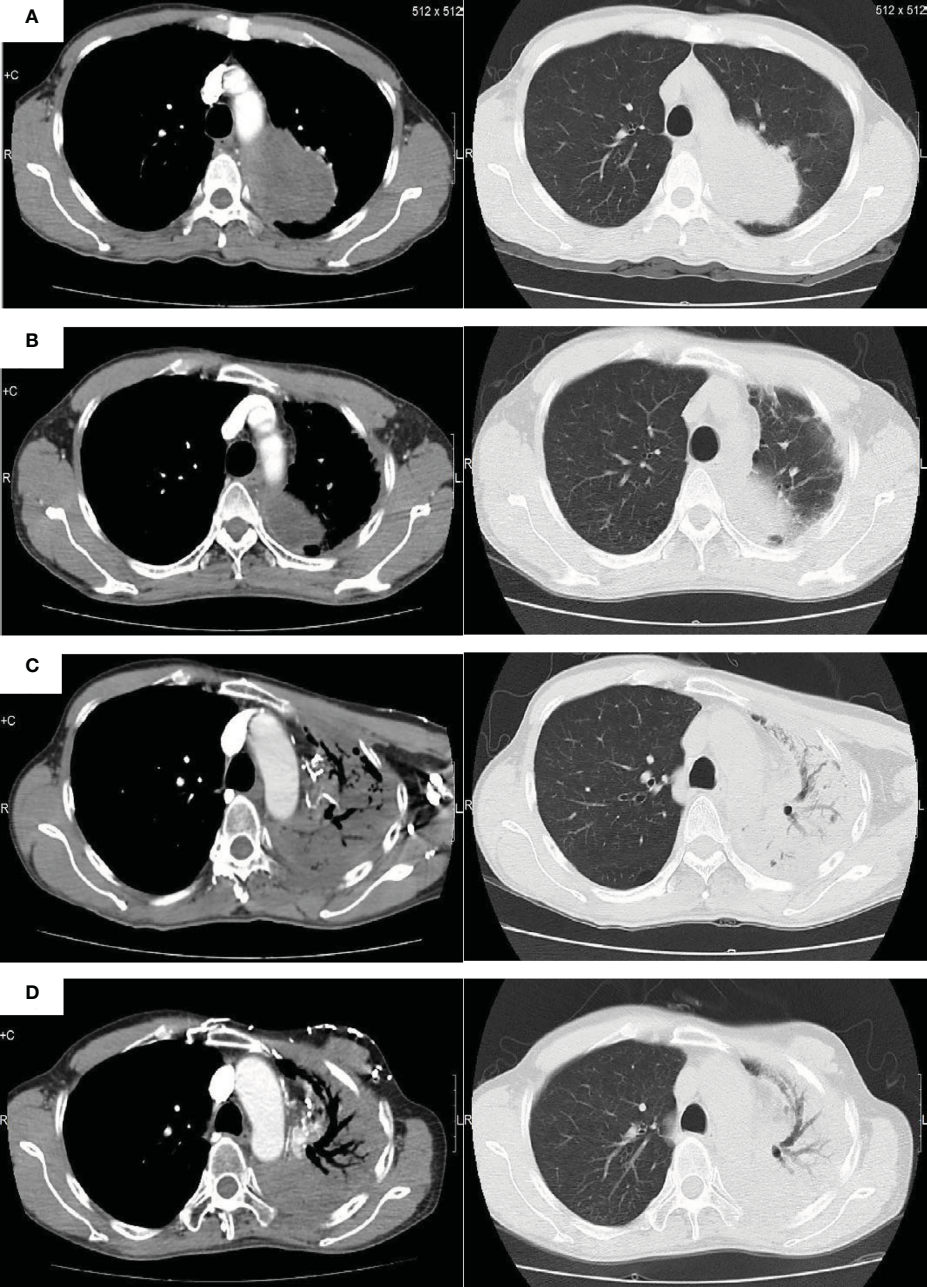
Radiologic images of the pulmonary blastoma patient. **(A)** Baseline image. **(B)** Image obtained following completion of first-line chemotherapy and radiotherapy. **(C)** Image obtained prior to anti-PD-1 immunotherapy. **(D)** Image obtained after anti-PD-1 immunotherapy.

The patient was a 50-year-old man. The patient’s father passed away due to tuberculosis and respiratory failure, while his mother remains in good health. The patient has two sisters and a half-brother, all of whom are healthy. At present, the patient does not have any children. He presented with chest tightness, chest and back pain, cough, blood in the sputum, and hypodynamia. The patient had a smoking history of 20 years before developing lung disease. Before the development of the disease, the patient had good health and no reported history of occupational exposure or genetic predisposition within the family. Contrast-enhanced computed tomography (CT) examination of the chest at presentation showed an upper left lung mass shadow (10.7 cm × 8.5 cm × 10.5 cm). The margin was poorly demarcated from the mediastinal pleura comprising multiple flaky low-density necrotic areas. The CT value ranged from 19 to 33 Hu. Whole-body positron emission tomography (PET)-CT scan revealed a mass hypermetabolic lesion in the upper left lung (SUV 8.5) and was considered upper left lung cancer. The lesion was infiltrated along the adjacent bronchial and pleural. And it was intricately related to the aortic arch with peripheral obstructive inflammation. Mild hypermetabolism (SUV 3.0) of left hilar and mediastinal lymph nodes (groups 5 and 6) reflected lymph node metastasis. No metastases were seen elsewhere throughout the body. Blood tumor markers (CEA, NSE, and CYFRA21–1) were normal. Examination of fine-needle aspiration biopsy of the lesion via CT revealed that tumor cells were spindle-shaped or oval, with diffuse infiltrative growth, necrosis of a small number of cartilage components, and loose interstitium. The tumor tissue consisted of both epithelial and mesenchymal tissues, with the epithelial component exhibiting features of low-grade adenocarcinoma. The mesenchymal component, on the other hand, exhibited characteristics of sarcoma with cartilage metaplasia, as illustrated in **Figure 1**. This was consistent with the diagnosis of PB. Immunohistochemistry revealed epithelial components: CK (+), TTF1 (+), CEA (–), CK7 focal (+), CK5/6 focal (+), S-100 focal (+), EMA focal (+); mesenchymal components: Vim (+), S-100 focal (+), Des small (+), CD99 individual (+), CD34 vascular (+), MC (-), Napsin A (-), and Ki-67 (30% +) (**Figure 2**). Due to the limited tissue sample available, certain immunotherapy predictors, including PD-L1, tumor mutation burden (TMB), and microsatellite stability (MSS), were not evaluated. The diagnosis was classic biphasic PB, and the clinical tumor stage was cT4N2M0 IIIB. The tumor could not be resected because the patient’s tumor was closely related to the aortic arch. Therefore, the multiple disciplinary teams (MDTs) believed that radical surgery could be performed. The first-line chemotherapy regimen included paclitaxel 175 mg/m^2^ (day 1) + cisplatin 25 mg/m^2^ (day 1–day 3), with a total of four cycles. Chest radiotherapy was given one month after chemotherapy at a dose of 75 Gy/35f, and efficacy was assessed as partial response (PR) after sequential chemoradiotherapy. Follow-up was repeated every three months following the first-line treatment, and PFS was 12.3 months. The patient reported worsening chest pain, with the chest CT showing that the lesion was progressing. The left upper lung lesion was markedly enlarged, and solid changes appeared. The patient experienced difficulty falling asleep due to chest pain, requiring Oxycodone Hydrochloride Controlled-release Tablets at a dosage of 360 mg q12 h to achieve a Numerical Rating Scale (NRS) score below 3. This pain level significantly impacted the patient’s ability to work and carry out daily activities. As a distinct subtype of NSCLC, classical biphasic PB (CBPB) poses challenges for second-line chemotherapy alone, and no known driver gene mutation is currently suitable for targeted therapy in this condition. Previous reports have demonstrated that PD-L1 expression was observed in roughly 12% of PPB patients (5). Furthermore, case studies have identified high PD-L1 expression in patients with CBPB (6). As such, the combination of chemotherapy and ICIs was considered a potential treatment approach. The treatment was changed to gemcitabine 1250 mg/m^2^ (d1, 8) and sintilimab 200 mg (d1). After one cycle of treatment, the patient developed third-degree neutropenia, a second-degree rash, and anorexia; we attributed these side effects to gemcitabine. Gemcitabine chemotherapy was discontinued at the patient’s insistence, and only sintilimab immunomonotherapy was administered. Over time, the patient’s chest pain gradually subsided, and they were able to resume daily activities. The dosage of Oxycodone Hydrochloride Controlled-release Tablets was consequently reduced to 80 mg q12 h. No significant adverse effects were observed during this treatment period. Based on imaging evaluations, the patient’s disease state remained stable, classified as stable disease (SD) (see **Figure 3**). The patient is currently receiving sintilimab for 27 months. His condition was stable, with a performance status (PS) score of 1. The patient has been alive for nearly 40 months and has a good quality of life.

## Discussion

PB is a rare subtype of lung tumor in humans and has been divided into three types of well-differentiated fetal adenoblastoma (WDFA), namely uniphasic PB, pleural PB (PPB), and CBPB. Of these, CBPB has been reported to be the most common (4). Since 2004, the World Health Organization’s (WHO’s) classification of lung tumors has classified CBPB as pulmonary sarcoma-like carcinoma along with pleomorphic carcinoma, spindle cell carcinoma, giant cell carcinoma, and carcinosarcoma (7, 8). The most recent WHO classification, released in 2021, distinguishes PB as a distinct entity from PPB (9, 10). PB is a rare form of sarcomatoid non-small cell lung cancer characterized by a biphasic pattern.WDFA is considered a histological variant of lung adenocarcinoma. PPB is classified as a soft tissue tumor, also known as childhood PB. PPB has been reported to almost exclusively occur in children and adolescents under the age of 14 years. It is characterized by predominantly localized growth, with certain cases more aggressive and metastatic.

Unlike PPB and WDFA, CBPB is a biphasic tumor and consists of fetal adenocarcinoma (usually low-grade) and the original mesenchymal stroma, which is more common in middle-aged individuals. CBPB usually presents with non-specific clinical manifestations similar to those of lung cancer, such as a large lump in the chest causing pain, hemoptysis, cough, and dyspnea. The absence of distinct hematologic tumor markers has made it challenging to diagnose this type of tumor. Consequently, immunohistochemical analysis is required for diagnosis due to its biphasic structure. Microscopic images of biphasic differentiation of primitive epithelial components and primitive embryo components have been observed. The epithelial component is positive for cytokeratin, CEA, epithelial membrane antigen (EMA), thyroid transcription factor-1 (TTF-1), and surface protein α staining, whereas matrix components are positive for vimentin, desmin, muscle-specific actin, myoglobin, and S-100 staining (11). Immature focal striated muscle and/or cartilage differentiation is observed in approximately 25% of tumors (12), whereas mulberry-like structures have been noted in 43% of CBPs, often during neuroendocrine differentiation. In the present case, the patient’s pathological cell morphology was naïve, with mulberry-like structure and cartilage components and neuroendocrine differentiation, which was consistent with the manifestations of PB.

Because CBPB is rare, only a few studies have explored long-term outcomes in these populations. Because the majority of studies have been case reports and literature reviews, information on prognostic factors is little. It has been reported that CBPB is an aggressive tumor with a relatively poor prognosis, whereas WDFA and PPB have a relatively good prognosis. Because CBPB has a higher probability of metastasis, its 5-year survival rate has been reported to be approximately 15%, compared with 62% for PPB and 75% for WDFA (13, 14). Xiang et al. reported that nearly half of PB patients achieved long-term survival (15), with 5- and 10-year survival rates for all PB patients being 58.2% and 48.5%, respectively. Even 40% of patients with metastatic PB achieved long-term survival beyond 5 years. However, two-thirds of patients with CBPB die within 2 years of diagnosis, with survival rates of only 16% and 8% at 5 and 10 years after diagnosis (16).

Although treatment strategies for PB include radiation therapy and chemotherapy, standard therapy has not been established because of the rarity of the tumor (17). Moreover, oncogenic driver gene mutations have been poorly studied, further limiting the treatment approach. It is reported that *DICER1* mutations could be important drivers (18). The β-catenin gene mutation is the most frequently observed molecular alteration in CBPB. In addition, mutations in p53, BRCA2, ERBB4, ALK, MET, BRAF, RAF1, PTEN, EGFR, and PIK3CA have also been documented in association with this disease (19). In a separate study, Taylor M. Jenkins et al. reported on the case of a CBPB patient with a ROS-1 rearrangement, which was the first such case to be documented. The patient’s tumor was subjected to fluorescence *in situ* hybridization (FISH), revealing a ROS-1 rearrangement in 7 out of 50 tumor cells (14%). The tumor tested negative for ALK and RET rearrangements. The patient was subsequently treated with crizotinib. However, due to metastasis of the disease at the time of treatment initiation, the patient’s condition continued to worsen, and they passed away 7 months after diagnosis (20). Surgical enlarged resection and lymphadenectomy are considered the treatment of choice. Nearly 80% of patients undergo surgery after diagnosis (21), whereas the preferred treatment for PB is enlarged resection plus lymphadenectomy. However, 43% of patients with tumors recur within 1 year of surgery and tend to metastasize to the brain, mediastinum, pleura, diaphragm, liver, heart, and soft tissues (22). Postoperative chemoradiotherapy could be considered in case of lymph node metastases or involvement of surrounding tissues. However, only a few cases are sensitive to radiation chemotherapy (23). For instance, Larsen et al. reported a chemotherapy response rate of 16% in 43 cases of classic biphasic PB. Postoperative adjuvant radiation therapy is considered if N2 lymph node metastasis is observed. Researchers in 1984 proposed certain chemotherapy drugs, including cyclophosphamide, vincristine, doxorubicin, and actinomycin. These agents have been reported to be effective in tumors that histologically resemble PB, such as nephroblastoma and rhabdomyosarcoma (24). Subsequently, a combination of ifosfamide and doxorubicin and radiation therapy resulted in a partial response in patients who relapsed after PB surgery (25). In a 16-year-old patient with stage IV with PB, chemotherapy with etoposide and cisplatin resulted in 1-year survival.

In another case report, a combination of carboplatin with paclitaxel and bevacizumab was shown to be effective (26). The effect of targeted drugs on PB is unclear. In this regard, sorafenib has been reported to be effective in treating a biphasic PB patient with renal metastases (27). *MET* mutations, especially jumping mutations in exon 14, are common in lung sarcomatoid carcinomas (about 22%). However, the incidence of *MET* mutations in PB is currently unclear and could be explored as a potential target in the future. Most of the above treatment options have been derived from case reports, and we lack a standard treatment established by randomized clinical trials. This could be related to the rarity of PB and the difficulty of conducting clinical trials.

Cancer treatment has been revolutionized by the advent of immunotherapy. However, the expression of programmed death ligand 1 (PD-L1) in PB patients and the efficacy of receiving PD-L1 inhibitors have rarely been reported. For example, in 2015, Joaquim Bosch-Barrera reported the first unresectable CBPB patient with high expression of PD-L1 (6). The patient achieved complete resection after neoadjuvant therapy with a combination of immunotherapy and chemotherapy. Alipour et al. detected the expression of PD-L1 in 25 PPB patients with a positive rate of approximately 12%. These patients could benefit from immune checkpoint inhibitor therapy (5). The cases we reported acquired stable disease (SD) after treatment with sintilimab as a second-line therapy and remained stable for up to 27 months without progression. Sintilimab is a recombinant fully human anti-PD-1 monoclonal antibody approved by the China Medical Products Administration on December 24, 2018. The efficacy of sintilimab in treating CBPB has not been previously reported. This case provides new evidence and novel options for immunotherapy for patients with PB. The disadvantage is that the patient’s biopsy tissue puncture conditions are limited, so *PD-L1*, tumor mutational burden (*TMB*), microsatellite stabilization (*MSS*), and other driver genes could not be detected.

## Conclusion

In this case report, we present the treatment outcome of a CBPB patient who received second-line therapy using the PD-1 inhibitor, sintilimab, resulting in a significant long-term survival benefit. While immunotherapy represents a promising treatment approach for solid tumors, there is limited evidence regarding its effectiveness in treating CBPB Therefore, our findings provide valuable new insights into the potential use of immunotherapy in CBPB treatment. It is important to note that due to the limited availability of tissue samples, we were unable to report on the gene status or PD-L1 expression status of the patient in question.

## Data availability statement

The original contributions presented in the study are included in the article/supplementary material. Further inquiries can be directed to the corresponding authors.

## Ethics statement

The studies involving human participants were reviewed and approved by Guangzhou Chest Hospital ethics committee. The patients/participants provided their written informed consent to participate in this study. Written informed consent was obtained from the individual(s) for the publication of any potentially identifiable images or data included in this article.

## Author contributions

JH, YX, and WC conceived the idea of the article. NS composed the manuscript and figure-making. AL, LL, and JZ supported the clinical data. CL provided the IHC test. All authors contributed to the article and approved the submitted version.

## References

[1] Dixit R Joshi N Dave L. Biphasic pulmonary blastoma: An unusual presentation with chest wall, rib, and pleural involvement. Lung India (2014) 31(1):87–9. doi: 10.4103/0970-2113.126002 PMC3960824 24669096

[2] Magistrelli P D'Ambra L Berti S Bonfante P Francone E Vigani A. Adult pulmonary blastoma: Report of an unusual malignant lung tumor. World J Clin Oncol (2014) 5(5):1113–6. doi: 10.5306/wjco.v5.i5.1113 PMC4259939 25493248

[3] Robert J Pache JC Seium Y de Perrot M Spiliopoulos A. Pulmonary blastoma: report of five cases and identification of clinical features suggestive of the disease. Eur J Cardiothorac Surg (2002) 22(5):708–11. doi: 10.1016/S1010-7940(02)00529-8 12414034

[4] Force S Patterson GA. Clinical-pathologic conference in general thoracic surgery: pulmonary blastoma. J Thorac Cardiovasc Surg (2003) 126(5):1247–50. doi: 10.1016/S0022-5223(03)00079-5 14665988

[5] Alipour Z Schultz KAP Chen L Harris AK Gonzalez IA Pfeifer J. Programmed death ligand 1 expression and related markers in pleuropulmonary blastoma. Pediatr Dev Pathol (2021) 24(6):523–30. doi: 10.1177/10935266211027417 PMC9196202 34266329

[6] Bosch-Barrera J Holguin F Baldo X Rubio M Porta R Fuentes R. Neoadjuvant chemoradiotherapy treatment for a classic biphasic pulmonary blastoma with high PD-L1 expression. Anticancer Res (2015) 35(9):4871–5. 26254381

[7] Franks TJ Galvin JR. Sarcomatoid carcinoma of the lung: histologic criteria and common lesions in the differential diagnosis. Arch Pathol Lab Med (2010) 134(1):49–54. doi: 10.5858/2008-0547-RAR.1 20073605

[8] Travis WD Brambilla E Nicholson AG Yatabe Y Austin JHM Beasley MB. The 2015 world health organization classification of lung tumors: Impact of genetic, clinical and radiologic advances since the 2004 classification. J Thorac Oncol (2015) 10(9):1243–60. doi: 10.1097/JTO.0000000000000630 26291008

[9] Tsao MS Nicholson AG Maleszewski JJ Marx A Travis WD. Introduction to 2021 WHO classification of thoracic tumors. J Thorac Oncol (2022) 17(1):e1–4. doi: 10.1016/j.jtho.2021.09.017 34930611

[10] Pooniya S McKinnie A Taylor T Will M Wallace W. Classic biphasic pulmonary blastoma (CBPB): a rare primary pulmonary malignancy. BMJ Case Rep (2021) 14(8):e244151. doi: 10.1136/bcr-2021-244151 PMC8356168 34376421

[11] Weissferdt A MC. Diagnostic pathology of pleuropulmonary neoplasia. Anticancer Res (2013) 33(5):2346. doi: 10.1007/978-1-4419-0787-5

[12] Travis WD. Sarcomatoid neoplasms of the lung and pleura. Arch Pathol Lab Med (2010) 134(11):1645–58. doi: 10.5858/2010-0086-RAR.1 21043818

[13] Le Caer H Teissier E Barriere JR Venissac N. Classic biphasic pulmonary blastoma: A case report and review of the literature. Crit Rev oncology/hematology (2018) 125:48–50. doi: 10.1016/j.critrevonc.2018.02.009 29650276

[14] Messinger YH Stewart DR Priest JR Williams GM Harris AK Schultz KA. Pleuropulmonary blastoma: a report on 350 central pathology-confirmed pleuropulmonary blastoma cases by the international pleuropulmonary blastoma registry. Cancer (2015) 121(2):276–85. doi: 10.1002/cncr.29032 PMC4293209 25209242

[15] Bu X Liu J Wei L Wang X Chen M. Epidemiological features and survival outcomes in patients with malignant pulmonary blastoma: a US population-based analysis. BMC Cancer (2020) 20(1):811. doi: 10.1186/s12885-020-07323-0 32847556 PMC7449001

[16] Brodowska-Kania D Kotwica E Paturej A Sosnicki W Patera J Gizewska A. What do we know about pulmonary blastoma?: review of literature and clinical case report. Nagoya J Med Sci (2016) 78(4):507–16. doi: 10.18999/nagjms.78.4.507 PMC5159477 28008207

[17] Lkhoyaali S Boutayeb S Ismaili N Aitelhaj M Ouchen F Benosman A. Neoadjuvant chemotherapy in well-differentiated fetal adenocarcinoma: a case report. BMC Res Notes (2014) 7:283. doi: 10.1186/1756-0500-7-283 24886749 PMC4017811

[18] de Kock L Bah I Brunet J Druker H Astigarraga I Bosch-Barrera J. Somatic DICER1 mutations in adult-onset pulmonary blastoma. Eur Respir J (2016) 47(6):1879–82. doi: 10.1183/13993003.00172-2016 27126690

[19] Zhao YY Liu L Zhou T Zhou NN Yang YP Hou X. A retrospective analysis of the clinicopathological and molecular characteristics of pulmonary blastoma. OncoTargets Ther (2016) 9:6915–20. doi: 10.2147/OTT.S117097 PMC5108598 27877056

[20] Jenkins TM Morrissette JJD Kucharczuk JC Deshpande CG. ROS1 rearrangement in a case of classic biphasic pulmonary blastoma. Int J Surg Pathol (2018) 26(4):360–3. doi: 10.1177/1066896917749928 29295663

[21] Knight S Knight T Khan A Murphy AJ. Current management of pleuropulmonary blastoma: A surgical perspective. Children (Basel) (2019) 6(8):86. doi: 10.3390/children6080086 31349569 PMC6721434

[22] Van Loo S Boeykens E Stappaerts I Rutsaert R. Classic biphasic pulmonary blastoma: a case report and review of the literature. Lung Cancer (2011) 73(2):127–32. doi: 10.1016/j.lungcan.2011.03.018 21513998

[23] Surmont VF van Klaveren RJ Nowak PJ Zondervan PE Hoogsteden HC van Meerbeeck JP. Unexpected response of a pulmonary blastoma on radiotherapy: a case report and review of the literature. Lung Cancer (2002) 36(2):207–11. doi: 10.1016/S0169-5002(01)00465-2 11955657

[24] Medbery CA 3rd, Bibro MC Phares JC Veach SR Martin JE Pasquale DN. Pulmonary blastoma. case report and literature review of chemotherapy experience. Cancer (1984) 53(11):2413–6. doi: 10.1002/1097-0142(19840601)53:11<2413::aid-cncr2820531108>3.0.co;2-e 6370414

[25] Lindet C Vanhuyse M Thebaud E Robin YM Penel N. Pulmonary blastoma in adult: dramatic but transient response to doxorubicin plus ifosfamide. Acta Oncol (2011) 50(1):156–7. doi: 10.3109/0284186X.2010.491087 20670092

[26] Sakata S Saeki S Hirooka S Hirosako S Ichiyasu H Kohrogi H. A case of biphasic pulmonary blastoma treated with carboplatin and paclitaxel plus bevacizumab. Case Rep Oncol Med (2015) 2015:842621. doi: 10.1155/2015/842621 26075125 PMC4444532

[27] Mulamalla K Truskinovsky AM Dudek AZ. Pulmonary blastoma with renal metastasis responds to sorafenib. J Thorac Oncol (2007) 2(4):344–7. doi: 10.1097/01.JTO.0000263719.76944.0a 17409808

